# Stigma and related factors among renal dialysis patients in China

**DOI:** 10.3389/fpsyt.2023.1175179

**Published:** 2023-07-31

**Authors:** Bing Li, Di Liu, Yue Zhang, Pengshi Xue

**Affiliations:** Department of Nursing, Shengjing Hospital of China Medical University, Shenyang, China

**Keywords:** renal dialysis, stigma, fear of progression, hope, social support

## Abstract

**Background:**

Stigma is an important psychological concept that is being studied in many diseases. However, there have been few studies on stigma in renal dialysis patients in China. This study aimed to investigate the level of stigma and its potential influencing factors among Chinese renal dialysis patients.

**Methods:**

A cross-sectional study was conducted among renal dialysis patients in two Chinese dialysis centers between April 2022 and July 2022. Two hundred four renal kidney patients were interviewed with a questionnaire on demographic variables using the Social Impact Scale (SIS), Resilience Scale-14 (RS-14), Herth Hope Index(HHI), Multidimensional Scale of Perceived Social Support (MSPSS), Revised Life Orientation Test(LOT-R), Perceived Stress Scale (PSS-4) and Fear of Progression (FoP). T-test/univariate one-way ANOVA, Pearson’s R, and hierarchical linear regression analysis were used to investigate the factors that influence stigma.

**Results:**

Renal dialysis patients in China experienced a moderate level of stigma (52.36 ± 8.16). Stigma was negatively correlated with resilience, hope, and perceived social support, whereas it was positively associated with perceived stress and fear of progression. However, it showed no significant relationship between optimism and stigma. Hierarchical linear regression analysis showed that hope (β = -0.318, *P* < 0.001), social support (β = -0.193, *P* < 0.01), perceived stress (β = 0.197, *P* < 0.01), and fear of progression (β = 199, *P* < 0.01) were found to be associated with stigma among the renal dialysis patients. All four variables in the model could explain 34.6% of the variance in stigma among renal dialysis patients in China.

**Conclusion:**

According to this study, renal dialysis patients in China face a moderate level of stigma. Stigma was found to be negatively related to hope and social support but positively associated with perceived stress and fear of progression. Future research on the stigma of renal dialysis patients should include hope-based interventions, proper and specific social support strategies, stress management interventions, and more disease-related information.

## 1. Introduction

End-stage kidney disease, which is the fifth stage of chronic kidney disease, is the most severe stage of chronic kidney failure caused by various factors (1). The patient’s renal function is completely or nearly completely lost, which seriously threatens the patient’s life (2). It was reported that the number of patients requiring renal replacement therapy ranged from 4.902 to 9.701 million worldwide and that this figure would more than double by 2030 (3). Similar conditions can be found in America (4), Europe (5), China (6), and other countries.

Renal dialysis is the most commonly used treatment for patients with end-stage kidney disease, with more than 90% receiving it. The therapy provides those patients with a potentially longer life span. However, the adverse effects of therapy include low quality of life (7), fatigue (8), sleep disorders (9), anorexia, nausea/vomiting, pruritus, sleepiness, difficulty concentrating, pain (10), which cannot be ignored. In addition to the physical effects, the psychological effects should be considered. It is reported that when patients decide on renal dialysis, they tend to avoid the therapy due to thinking that dialysis is the most stressful part of the disease (11). In addition to distress (12), loss (13), well-being (14), negative coping (15), anxiety (16), depression (17), and so on cannot be ignored as patients on dialysis. Many studies have found that renal dialysis patients’ experience feeling of passivity and restriction (18). They can hardly do their original jobs anymore (19), and their lives must be entirely re-planned to adapt to dialysis. Dialysis patients have low self-esteem, believe they are a burden on their family members and do not contribute to the family. They expose themselves to uncertain future and are hesitant to interact with others (20). These changes are visible and life-long and may result in the absence of individuals from full social acceptance, and patients themselves may have thoughts of escaping society, which corresponds to the concept of stigma.

Stigma describes a deeply discreditable attribute or characteristic, conveying a spoiled social identity and a sense of disgrace in a particular social context, disqualifying the individual from social recognition (21). Stigma is a psychological stress response. Patient’s self-assessment plays a crucial role in the production and formation of stigma (22, 23). Stigma increases the psychological burden of patients, causing them to be labeled, stereotyped, isolated, lose their status, and even face discrimination (24). It will affect the patient’s quality of life and follow-up treatment and even lead to adverse consequences such as social escape and suicide (25). Studies have also shown that stigma reduces self-esteem, self-efficacy, and belief in own abilities (26). Stigma has widely been used in patient populations such as cancer (27), chronic diseases (28), psychiatric disorders (29), addiction diseases (30), obesity (31), and geriatric diseases (32), and so on to provide many new perspectives and findings.

Stigma is an important concept in psychology and has been a research topic for many diseases. Surprisingly, little information is currently available about stigma in renal dialysis patients. Considering such immense pressure and its effect on renal dialysis patients and its importance, stigma should be approached from different perspectives. Therefore, the present study aimed to fill this knowledge gap. Studies examining the stigma associated with other diseases have revealed that, in addition to demographic and clinical characteristics, resilience (33), optimism (34), social support (35, 36), hope (37), perceived stress (38) were all related factors to stigma. Furthermore, renal dialysis was a life-long treatment with multiple complications. Many patients in the chronic kidney disease stage reported their fear of disease progression (39). Excessive fear of disease progression may cause patients to label the disease even more and devalue themselves. Thus, we would explore the relationship of the aforementioned factors in renal dialysis patients from the Chinese population. The hypothesis proposes that stigma is positively associated with stress and fear of progression and negatively associated with resilience, hope, social support, and optimism among renal dialysis patients. Accordingly, we will test this hypothesis in the current study. This study aimed to investigate the level of stigma and its potential influencing factors among Chinese renal dialysis patients. We hope that the findings of our study, particularly the identification of stigmatizing factors, will be useful and shed new light on the management of renal dialysis patients.

## 2. Materials and methods

### 2.1. Study settings

This is a cross-sectional designed study that was carried out at two renal dialysis centers in China. Data was collected between April 2022 and July 2022. The Ethics Committee of China Medical University approved this study (2022PS153K).

### 2.2. Subjects

Patients receiving renal dialysis therapy who understood and completed the questionnaire were invited to participate in the study under their consent, while patients in severe conditions were excluded. The study size was arrived at following the below formula: $n=\frac{z_{\alpha }^{2}\sigma^{2}}{{}^{\delta^{2}}}$. The parameters in the study were set as follows: α = 0.05, Zα = 1.96, σ = 10.58 (which was arrived via pre-test), δ = 1.5; therefore, n = 1.962*10.58^2^/1.52 = 191.1. The sample size was increased by 10%∼20%, considering invalid questionnaires, resulting in a final sample size of 211∼230.

### 2.3. Data collection

The entire research process was anonymous, and the patients were voluntary. The researchers of the study uniformly trained the five investigators. After the patients agreed to participate, the paper questionnaires were filled out in a separate and undisturbed space in the hospital to prevent patients from influencing each other while filling out questionnaires. The investigator is responsible for interpreting the questionnaire items without any incentive. Another trained investigator performed quality control on site. Epidata software (version 3.1) was used for data entry and review.

### 2.4. Tools

Questionnaires including demographic and clinical characteristics were self-developed in the study. The demographic section included age, gender, education level, job status, religious belief, income, family structure, and medical payments. Also, the clinical variables section included the approach and the course of dialysis of the patient (how long the dialysis lasted).

#### 2.4.1. Stigma

The stigma of the respondents was measured using the Social Impact Scale (SIS) (40). SIS consists of 24 items divided into four categories: social rejection, financial insecurity, internalized shame, and social isolation. Each scale item has a four-point scale, with a total score ranging from 24 to 96. Cronbach’s α of stigma was 0.871 in this study.

#### 2.4.2. Resilience

Resilience was assessed using the Resilience Scale-14 (RS-14) (41). The RS-14 consists of 14 items on a 7-point scale, with an overall score ranging from 14 and 98. In the current study, Cronbach’s α of resilience was 0.863.

#### 2.4.3. Hope

The level of hope was assessed by the Herth Hope Index (HHI) (42). The HHI consists of 12 items, and each item is scored on a 4-point scale. The total HHI score ranges from 12 to 48, and a higher total score reflects a higher level of hope. In the present study, Cronbach’s α of hope was 0.866.

#### 2.4.4. Social support

The Chinese version of the Multidimensional Scale of Perceived Social Support (MSPSS) was used to assess perceived social support (43). The MSPSS comprises ten items scored on a 7-point scale; the total score ranges from 12 to 84, with a higher score indicating more social support. Cronbach’s α of social support was 0.935 in the present research.

#### 2.4.5. Optimism

The 10-item Revised Life Orientation Test (LOT-R) was used to assess optimism (44). The LOT-R uses a 5-point rating system. A higher score indicates a higher level of optimism. Cronbach’s α of optimism was 0.621 in this research.

#### 2.4.6. Perceived stress

The 4-item Perceived Stress Scale (PSS-4) was used to assess perceived stress (45). PSS-4 is scored using a 5-point scale, with a total score ranging from 0 to 16. Higher scores indicate a higher level of perceived stress. Cronbach’s α of perceived stress was 0.764 in this study.

#### 2.4.7. Fear of progression

The Fear of Progression Questionnaire-Short Form (Fo P-Q-SF) was used to assess the Fear of Progression (FoP) (46). The Fo P-Q-SF is a 12-item scale with a 5-point rating and a total score ranging from 12 to 60. A higher score indicates a greater fear of disease progression. Cronbach’s α for fear of progression was 0.895 in the present study.

### 2.5. Statistical analyses

Data analysis was performed using the statistical software package for social sciences (SPSS 20.0). The significance for all statistical tests was 0.05 (2-tailed). Each continuous variable is first tested for normality and homogeneity of variance. Independent-samples t-tests and one-way ANOVA were used to describe the distribution of stigma for categorical demographic and clinical variables in renal dialysis patients. Pearson’s R-test was used to assess the correlations between resilience, hope, social support, optimism, stress, fear of progress, and stigma. Hierarchical linear regression analysis was used to assess the research hypotheses. To avoid overfitting the regression model, the one-way ANOVA/t-test variable with *P* < 0.2 was entered as the control variable in the first step of the hierarchical regression analysis (47). Then, the independent variables (resilience, hope, perceived social support, perceived stress, fear of progress) also entered the second step of the hierarchical regression. Diagnostic tests for multicollinearity were performed using tolerance and variance inflation factor (VIF). The data provided by the regression model include standardized regression coefficient (β), R^2^, adjusted R^2^ (Adj. R^2^), R^2^-change, and F value.

## 3. Results

### 3.1. Descriptive statistics

A total of 230 questionnaires were distributed in this study. Twenty patients refused to participate in the survey, and six invalid questionnaires. There were 204 valid questionnaires with an effective response rate of 88.7%.

Out of the 204 respondents, 126 (61.8%) were male, and 78 (38.2%) were females. Nearly half of them (46.1%) were above 60 years old. All the patients had medical insurance. Only 12 (5.8%) patients had a regular employee. In terms of clinical variables, most respondents (96.1%) used an autogenous arteriovenous fistula to access dialysis. More than half of the respondents had dialysis for less than five years. Table 1 presents the details.

**TABLE 1:** Demographic and clinical characteristics and the level of stigma among renal dialysis patients (*n* = 204).

Variables	*N* (%)	Mean (SD)	T/F	*P*
Gender			0.463	0.644
Male	126(61.8)	52.57 (7.22)		
Female	78 (38.2)	52.03 (9.54)		
Marriage			0.345	0.731
Single/divorced/ widow	47 (23.0)	52.72 (9.98)		
Married/ cohabitation	157 (77.0)	52.25 (7.57)		
The course of dialysis in the patient (Year)			3.036	0.050
<5	104 (51.0)	51.05 (8.71)		
5∼10	70 (34.3)	53.37 (7.53)		
≥10	30 (14.7)	54.57 (6.96)		
Age of patients			0.078	0.925
≤40	27 (13.2)	52.81 (8.36)		
41–60	83 (40.7)	52.13 (8.08)		
>40	94 (46.1)	52.44 (8.26)		
Education of patients			0.256	0.775
Middle school or lower	82 (40.2)	52.63 (9.45)		
High school or secondary school	60 (29.4)	52.63 (5.84)		
College or university or above	62 (30.4)	51.74 (8.34)		
Job status			0.293	0.746
Unemployed	178 (87.3)	52.53 (8.22)		
Regular employee	12 (5.8)	51.00 (6.95)		
Temporary employee	14 (6.9)	51.43 (8.75)		
Religious belief			1.818	0.071
No	196 (91.3)	52.57 (8.17)		
Yes	68 (8.7)	47.25 (6.48)		
Income (RMB, yuan)			3.204	0.043
<3,000	51 (25.0)	52.86 (8.38)		
3,000–6,000	117 (57.4)	53.09 (8.76)		
>6,000	36 (17.6)	49.28 (4.46)		
Family structure			1.483	0.229
Live with unmarried child (ren)	32 (15.7)	54.34 (9.16)		
Live with married child (ren)	57 (27.9)	51.25 (8.28)		
Live alone/or with spouse	115 (56.4)	52.37 (7.77)		
Smoking			0.671	0.513
No	139 (68.1)	52.19 (8.77)		
Yes	57 (28.0)	51.67 (7.00)		
Stopped smoking	8 (3.9)	56.75 (2.87)		
Drinking			1.129	0.261
No	176 (86.3)	52.73 (8.24)		
Yes	28 (13.7)	50.07 (7.67)		
Approach of dialysis			0.580	0.561
Autogenous arteriovenous fistula	196 (96.1)	52.24 (7.92)		
Long- term deep vein catheterization	6 (2.9)	55.83 (15.56)		
Temporary catheterization	2 (1.0)	53.50 (3.54)		

*N*, number.

### 3.2. Stigma level

Table 2 depicts the level of stigma and its dimensions among renal dialysis patients.

**TABLE 2:** The levels of stigma among renal dialysis patients (*N* = 204).

Variables	Items	Mean ± SD	Actual scoring range	Average item score
Social rejection	9	19.09 ± 3.51	9∼30	2.12
Financial insecurity	3	6.13 ± 1.40	3∼9	2.04
Internalized shame	5	11.83 ± 2.14	7∼17	2.37
Social isolation	7	15.31 ± 2.90	7∼23	2.19
Stigma	24	52.36 ± 8.16	27∼79	2.18

### 3.3. Correlation among continuous variables

Table 3 depicts the correlation analysis results of resilience, hope, perceived social support, optimism, perceived stress, fear of progression, and stigma among renal dialysis patients. Stigma was negatively correlated with resilience (*r* = -0.386, *P* < 0.001), hope (*r* = -0.448, *P* < 0.001), perceived social support (*r* = -0.393, *P* < 0.001), and positively associated with and perceived stress (*r* = 0.255, *P* < 0.001), fear of progression (*r* = 0.314, *P* < 0.001). Besides, it showed no significant relationship between optimism and stigma (*P* > 0.05).

**TABLE 3:** Descriptive statistics and correlations in continuous variables among renal dialysis patients (*N* = 204).

	Means	SD	Stigma	Resilience	Hope	Social support	Optimism	Perceived stress
Resilience	68.55	12.93	-0.386***	1				
Hope	35.00	4.54	-0.448***	0.471***	1			
Perceived social support	64.92	12.15	-0.393***	0.534***	0.540***	1		
Optimism	15.28	2.67	-0.124	0.209**	0.397***	0.337***	1	
Perceived stress	6.83	3.08	0.255***	-0.168*	0.033	0.031	-0.340***	1
Fear of Progression	31.50	9.69	0.314***	-0.256***	-0.086	-0.057	0.000	-0.354***

*P < 0.05; **P < 0.01; ***P < 0.001 (two-tailed).

### 3.4. Hierarchical linear regression analysis

The influencing factors of stigma in renal dialysis patients were investigated using hierarchical linear regression analysis. Multiple regression analysis included variables significantly related to stigma in univariate analysis and variables related to the psychological status of renal dialysis patients. This study included demographic variables (the course of dialysis in the patient, religious belief, and income), resilience, hope, perceived social support, perceived stress, and fear of progression in the regression analysis. Hope (β = -0.318, *P* < 0.001), social support (β = -0.193, *P* < 0.01), perceived stress (β = 0.197, *P* < 0.01), fear of progression (β = 199, *P* < 0.01) were associated with stigma in renal dialysis patients, with all four variables in the model explaining 34.6% of the variance in stigma in renal dialysis patients. There is no collinearity between the variables (Tolerance > 0.5, VIF < 2). Table 4 lists the details.

**TABLE 4:** Hierarchical linear regression analysis on stigma among renal dialysis patients (*N* = 204).

Variables	Resilience					
	**β**	***P***	**β**	***P***	**Tolerance**	**VIF**
**Step 1**						
course of dialysis						
<5(reference)						
5 10	0.140	0.054	0.114	0.058	0.903	1.107
ł10	0.139	0.056	0.089	0.142	0.885	1.131
Income						
<3,000 (reference)						
3,000–6,000	0.014	0.861	0.039	0.576	0.677	1.487
>6,000	-0.162	0.048	-0.092	0.184	0.673	1.485
**Religious belief**	-0.105	0.129	-0.075	0.197	0.956	1.046
**Step 2**						
Hope			-0.318	0.000	0.702	1.424
Social support			-0.193	0.005	0.690	1.449
Perceived stress			0.197	0.002	0.838	1.193
Fear of Progression			0.199	0.001	0.846	1.182
F	3.070*		14.421***			
R^2^	0.058		0.372			
adjR^2^	0.039		0.346			
R^2^-change	–		0.314			

***P < 0.001 (two-tailed); *P < 0.05 (two-tailed).

## 4. Discussion

### 4.1. Stigma levels among renal dialysis patients

There have been a few studies on stigma in dialysis patients. We found that the level of stigma in the study was higher (48) than in previous studies of renal dialysis patients in limited studies. We speculate that this is due to age differences in the target population. Furthermore, we found that the level of stigma among renal dialysis patients was lower than that of some cancer patients (49–51), which could be attributed to the age and job status of patients in the study. In this study, nearly half of the patients (46.1%) were above 60 years old, and only 12 (5.8%) were regular employees. The conditions mentioned above may weaken the social stigma of renal dialysis patients. However, it does not mean that stigma on renal dialysis patients is insignificant. A recent study in Japan quantitatively elucidated dialysis-related stigma in patients on dialysis (52). Renal dialysis patients are subjected to long-term continuous treatment that may last until the end of their lives, disrupting their routines, social interactions, quality of life, mental health, and family life (7) as stigma is impossible to avoid. Some researchers reported that patients with dialysis (53) and chronic kidney disease (54–56) had an unspoken stigma, reminding us that we should pay more attention to stigma among patients with this disease. In terms of dimensions, we found that internalized shame scored highest. Previous studies showed that stigma could be most harmful when internalized (57), which could devalue themselves (58). Besides, the most stigma dialysis patients experienced were internalized shame and social isolation, which were consistent with patients with COPD (59) and diabetes (60). This implies that it is critical to change patients’ inner beliefs, values, idea and give them support in order to reduce the stigma associated with renal dialysis patients.

### 4.2. Factors associated with stigma among renal dialysis patients

In the present study, hope, social support, perceived stress, and fear of progression were potentially related to stigma among renal dialysis patients.

According to the results of hierarchical linear regression analysis, hope may have the strongest effect on stigma among renal dialysis patients, similar to previous studies on patients with other diseases (37, 61). It has been reported that hope is related to almost all health outcomes (62) for two reasons. Rather, hope is a vital positive psychological variable. Hope is a dynamic life force to expect a good future when facing uncertainty (63). Patients with a high level of hope have a promising attitude to the disease, which is beneficial to avoid devaluing themselves. Conversely, hope has been reported to have a positive effect on resilience (64), quality of life (65), stress (66, 67), anxiety (66), and depression (66) which may reduce the level of stigma indirectly. Furthermore, interventions based on Snyder’s hope theory have been reported effective in reducing the stigma level (68, 69). Therefore, we can take interventions based on hope of reducing the level of stigma in patients.

In the study, perceived social support was another variable that had a positive effect on decreasing the level of stigma among renal dialysis patients; a similar condition has also been found in previous related studies (70, 71). Social support is a vital strength for the patients. It is important to have a high level of social support due to the long-term, uninterrupted nature of the disease. And previous studies have also shown that social support had an important effect in deciding whether patients with end-stage kidney disease should receive dialysis (72). In the literature, social support is divided into instrumental support and emotional support (73). The supports mentioned above were both critical to the patients. However, some studies suggest that dialysis patients’ personal views about their illness can provide insight into whether patients could benefit from support (18, 74). It reminds us that we should pay attention to the thinking, and value of patients. It is in line with some research about social support, which has shown that social support works through hope and resilience (75). Given the preceding discussion, we should focus on using personalized combined with group intervention for dialysis patients in future work to improve the level of social support of patients. The content of the intervention is comprehensive, and the content of the intervention is what the patients need.

It was not surprising that perceived stress was an essential factor in the stigma among renal dialysis patients confirmed in previous studies on other diseases (76, 77). The levels of perceived stress are not the real level of stress but rather the stress that the patient perceives as an event. For the same event, different people may have different stress. The right amount of pressure is beneficial. However, if the patient’s stress perception level is excessive, it means that the disease has a significant impact on them. They usually look at the disease negatively and even look at themselves negatively. The patients may not believe in their future and themselves. The feelings mentioned above may make them more shamed. In this case, the stigma is more likely to arise. Not to mention that the severe disease was taboo and easily associated with uniformed and misinformed social impressions (78). Therefore, managing stress and maintaining it is a crucial issue. A study about stress management training has shown that stigma was reduced after the training (79), suggesting that stress management interventions can be implemented in dialysis patients.

Finally, fear of progression was identified as a significant potential influencing factor of stigma in dialysis patients. The fear of progression has been reported in patients with chronic kidney disease (39) without data on renal dialysis patients. Fear of progression (FoP) is a feeling of worry and fear caused by disease and its treatment that is different from traditional psychological dysfunction (80). The fear of progression in disease has been proved to related to quality of life (81), social function (82), happiness (83), well-being (84), and so on. Patients who are afraid of disease progression are unable to recognize and accept it. Even minor changes in illness can cause emotional panic. The abovementioned factors are detrimental to patients and would cause them to undervalue themselves. For renal dialysis patients, the possibility of disease cure is low. And, to some extent, the development of the disease in a negative direction is known. A high level of fear during the disease development process will make the patient more reluctant to reveal to others and make the patient look down on himself. It will also harm the patient’s treatment and quality of life. In the previous studies, group-based intervention (82) and illness perception (85) have been reported to be effective. Thus, actions and interventions aimed at increasing renal dialysis patients’ knowledge of disease-related information aided in the formation of a good group intervention.

However, in the study, some results were inconsistent with our hypotheses, such as optimism and resilience showed no significant relationship. Therefore, the exact mechanism of action of these two variables still needs further research.

## 5. Strength and limitations

This study aimed to identify potential factors related to stigma in renal dialysis patients. In this regard, our research provided some new information. The results showed that stigma in kidney dialysis patients were associated with hope, social support, perceived stress, and fear of progression. It emphasizes the significance of changing patients’ inner beliefs, values and ideas. Future work to reduce stigma among renal dialysis patients should include hope-based intervention, proper and specific strategies to improve social support, stress management interventions, and more disease-related information. This result indicated that stigma should be a major focus when dealing with renal dialysis patients.

Causation could not be established in this study due to the cross-sectional design. Future studies should assess whether the intervention can reduce stigma levels in renal dialysis patients. Furthermore, we focused only on the associations between stigma and resilience, hope, perceived social support, optimism, perceived stress, and fear of progress, whereas other factors that might affect stigma have been disregarded. Moreover, larger samples are required to improve representativeness. And the number of questions may limit the quality of the responses. The last but not the least, stigma is multifaceted in nature, dialysis patients experience stigma for multiple reasons, thus additional qualitative studies could be explored in the future research. Despite some limitations, our study provides important new information on stigma in renal dialysis patients with useful clinical implications.

## 6. Conclusion

According to this study, renal dialysis patients in China face a moderate level of stigma. Stigma was found to be negatively related to hope and social support but positively associated with perceived stress and fear of progression. Future research on the stigma of renal dialysis patients should include hope-based interventions, proper and specific social support strategies, stress management interventions, and more disease-related information.

## Data availability statement

The raw data supporting the conclusions of this article will be made available by the authors, without undue reservation.

## Ethics statement

The studies involving human participants were reviewed and approved by Committee of China Medical University (2022PS153K). The patients/participants provided their written informed consent to participate in this study.

## Author contributions

BL and PX were responsible for conception and design of the study. BL, DL, and YZ performed data extraction. BL did the data analysis and wrote the manuscript. PX contributed to the revision of the manuscript. All authors have reviewed the manuscript and given final approval of the version to be published.
